# Comparative Cost-Effectiveness of Drugs in Early versus Late Stages of Cancer; Review of the Literature and a Case Study in Breast Cancer

**DOI:** 10.1371/journal.pone.0146551

**Published:** 2016-01-22

**Authors:** Evgeni Dvortsin, Judith Gout-Zwart, Ernst-Lodewijk Marie Eijssen, Jan van Brussel, Maarten J. Postma

**Affiliations:** 1 Department of Pharmacy, Unit of PharmacoEpidemiology and PharmacoEconomics (PE2), University of Groningen, Groningen, The Netherlands; 2 University Medical Center Groningen (UMCG), Institute of Science in Healthy Aging & healthcaRE (SHARE), University of Groningen, Groningen, The Netherlands; Instituti Ospitalieri di Cremona, ITALY

## Abstract

**Background:**

Many oncological drugs that are being used in the adjuvant setting were first submitted for reimbursement in the metastatic stage, with differences in incremental cost-effectiveness ratios (ICERs) in both settings having potential implications for reimbursement and pricing. The aim of this study is to identify a possible trend in the cost-effectiveness for the early/adjuvant and late/metastatic stages of oncological drugs through review and case study.

**Methods:**

We reviewed pairs of cost-effectiveness analyses of the same oncological drug in different stages for Scotland and the Netherlands. The case study in this report was directed at trastuzumab in the Dutch situation. Using a simplified Markov model, the cost-effectiveness in early and late stage of breast cancer was calculated and compared to the findings from the review.

**Results:**

Comparable studies were found for cetuximab, bortezomib and bosutinib. Treatments in the late stage were found to be more expensive per QALY by a factor ranging from 1.5 to 12. The case study provided a similar result; late stage treatment was more expensive by a factor 10. Using, for example, a threshold of €80,000/QALY, the early stage of cetuximab, bosutinib and trastuzumab are deemed cost-effective, while their compared late stage is lifted over the threshold and potentially considered not cost-effective.

**Conclusion:**

ICERs of oncological drugs used in different stages are more unfavourable in the late stage than in the early stage. Applying a reasonable threshold may result in early stage treatment being deemed cost-effective while late stage potentially not. Authorities should be aware of this when assessing oncological drugs and interpreting the corresponding ICERs, in the situation where oncological drugs are generally most submitted for reimbursement in the late stage initially.

## Introduction

Cost-effectiveness is an increasingly important aspect in the reimbursement of new drugs, such as oncological drugs. This class of drugs is sometimes viewed as relatively expensive and specific policies have been developed for their reimbursement, such as the “Policy Rule on Expensive Drugs” in the Netherlands and “End-of-life drugs” exception from the cost-per-QALY (quality-adjusted life year) threshold for the UK [1, 2]. Some of these drugs took decades to develop, are highly innovative and require complex manufacturing processes, possibly and potentially justifying relatively higher pricing. Depending on the country, there are further special arrangements concerning these drugs, such as patient access schemes, price negotiations, conditional reimbursement and agreements on volume being made by the pharmaceutical industry, its cooperating partners and the government [3]. As said, one of the major issues in the reimbursement is cost-effectiveness. Many countries, including the Netherlands, take cost-effectiveness explicitly into consideration when evaluating reimbursement. Unlike for example the UK, The Netherlands have no formal cost-per-QALY threshold although €80,000 is regularly mentioned for drugs [4]. In the present situation, many oncological drugs that are being used in the adjuvant setting are also registered for the metastatic phase, and cost-effectiveness for reimbursement has been evaluated for both settings. This warrants for an interesting comparison of cost-effectiveness in both settings for the same drugs and questioning whether trends over various drugs could be identified.

A priori, we postulated that as an overall trend cost-effectiveness would tend to be more favorable in the early phase of the adjuvant setting than in the late phase of metastatic treatment. The former might potentially be related to better utilities and higher efficacies, thus benefiting a more positive cost-effectiveness outcome for the early phase. In addition, treatment costs in the early stage could be lower, with potentially lower dosing being sufficient. Yet, resource consumption could be again higher as the overall survival tends to be longer, prolonging the duration of treatment for these patients. Ergo, an analytical approach is warranted to substantiate our a-priori hypothesis. In this study, we make an effort to identify a consistent trend in the cost-effectiveness of various specialist oncological drugs, in the adjuvant and metastatic phases. The first part of the paper will be targeted to reviewing pairs of cost-effectiveness analyses concerning the same oncological drug, but in different phases of illness; i.e., early vs. late. In addition a model is created that specifically looks at trastuzumab as an example; i.e., the monoclonal antibody that targets against the extracellular domain of the (HER2)-positive breast cancer patients, investigated in various clinical trials [5–7]. All in all, the aim of the integrated study is to provide a possible trend in the cost-effectiveness for the early/adjuvant and late/metastatic phases of oncological drugs.

## Methods

### Review

For the review part of our paper, cost-effectiveness studies were searched on oncological drugs used in different stages of cancer. Notably, pairs of comparable studies—one for early stage and one for late stage—were searched for similar (or ideally the same) countries, using a combination of search terms including [cost-effectiveness], [cancer], [oncology], [treatment], [ICER] and [drug], excluding any non-pharmacoeconomic publications. No time limitations were set; yet knowing that cost-effectiveness is only relevant in decision making since the turn of the century, we would expect most analyses beyond approximately 2005. Countries addressed here involve the Netherlands and Scotland; with both countries involved having relatively similar systems of reimbursement and clinical recommendations [8]. To assess the cost-effectiveness, authorities in both countries only use phase III clinical studies, as pharmacoeconomic evaluation is done at or soon after registration of new drugs, typically in the absence of observational data. Other similarities concern: obligations for manufacturers to hand in cost-effectiveness analyses for new drugs, explicit roles for cost-effectiveness studies in the process of recommendations, no formal threshold for cost-effectiveness and publicly available information on these matters. Also, the Netherlands and Scotland are comparably early with their drug assessments, whereas other countries often review submissions later, with potentially data already being available for a cost-effectiveness analysis. For example, in assessments of UK’s NICE, preferably observational data are included.

Often the studies identified were performed for the specific purpose of reimbursement. Studies were searched on the websites of the authorities involved [1, 9]; i.e.:

- Scottish Medicines Consortium (SMC); and
- The National Health Care Institute (“Zorginstituut Nederland” or ZiNL; former Dutch Foundation for Health Care Insurance or “College voor Zorgverzekeringen” or CVZ) and its Drugs Committee (“Commissie Geneesmiddelen”; former Committee Pharmaceutical Help or “Commissie Farmaceutisch Hulp”).

Notably, for oncological drugs, these bodies sometimes apply slightly deviating and specific methods, although the overall approach is grossly the same as for other types of drugs. For example, the Dutch authorities had a specific policy rule in force for some time concerning oncological (and other expensive) hospital drugs. Upfront, trastuzumab in early breast cancer (HERA clinical trial [6]) and late breast cancer (M77001 study group [7]) and bortezomib in multiple myeloma—with both early and late indications being analyzed by SMC [10,11]—were seen as role models for our approach.

Searches on the authorities’ websites were done alphabetically. The economic evaluations of both phases were compared to each other, with specific attention to the assumptions concerning:

- Dosing and duration of treatment
- Efficacy and safety
- Costs
- Utilities underlying the QALYs; and
- Transition probabilities.

### Case study

A case study was included for illustrative purposes only and definitely not intended to provide a full-fledged novel stand-alone analysis. The case study was directed at trastuzumab in the Dutch situation. Trastuzumab is a monoclonal antibody that targets against the extracellular domain of the (HER2)-positive breast cancer patients. A Markov model was developed to analyze the cost-effectiveness in adjuvant and metastatic stages. For both stages, situations with trastuzumab were compared to standard-of-care (SOC). The model was based on the specific trial data gathered in the adjuvant and metastatic phases [6, 7]. The costs and adjusted utilities were taken from the report of the manufacturer of trastuzumab [12].

**Fig 1:**
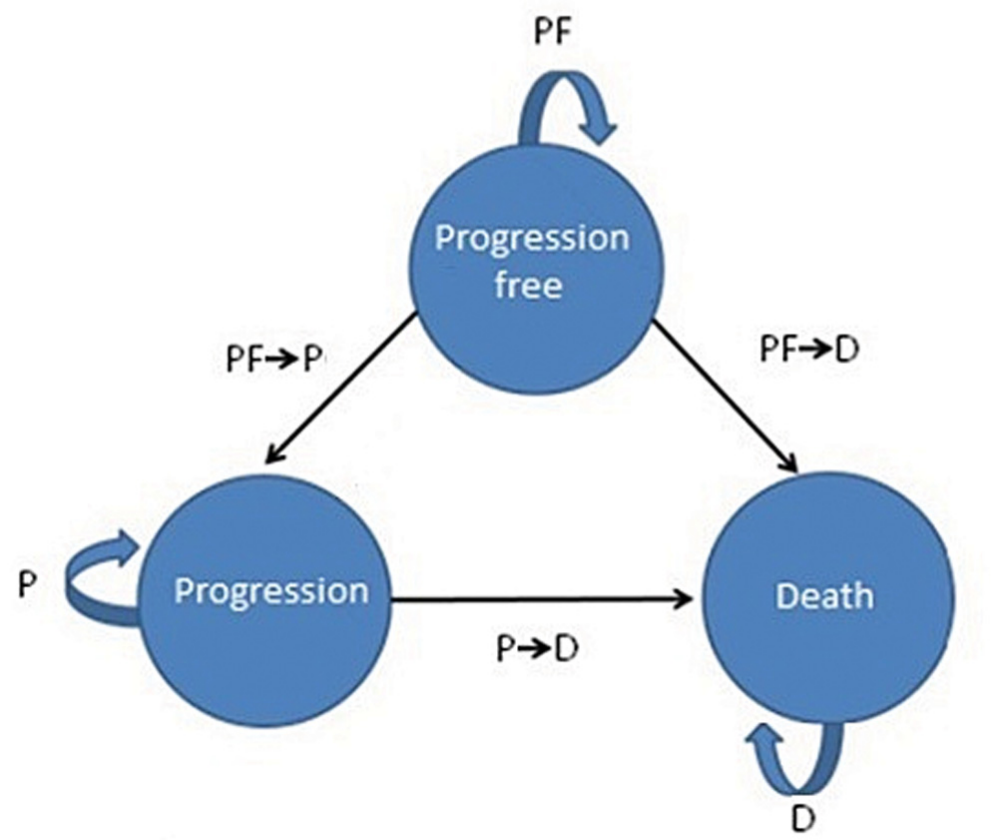
The Markov model with transition probabilities. PF: Progression-free survival, P: Progression, D: Death, PF-> P: Progression-free to progression, PF-> D: Progression-free to death, P->D: Progression to death.

The Markov model, as shown in Fig 1, includes a hypothetical cohort of 1000 patients followed in a lifetime simulation until the age of 100 years. For the early stage analysis, these 1000 patients start in the progression free (PF) stage, while in the late phase analysis these 1000 patients start in the progression (P) stage. The mean age of the patients was 51 years, corresponding to a simulation of 49 years [6, 12].

For the early stage, data such as annual transition probabilities were based on Smith et al. (2007), where trastuzumab’s effect on overall survival in Belgian patients with HER2+ breast cancer was investigated after a median follow-up of 2 years in the Herceptin Adjuvant (HERA) study [6]. HERA is an international multicenter randomized trial that compared 1 year or 2 years of trastuzumab treatment with observation alone after standard neoadjuvant or adjuvant chemotherapy in patients with HER2+ adjuvant breast cancer. Trastuzumab was administered every 3 weeks in a maintenance dose for 1 or 2 years. Results showed that the unadjusted hazard ratio (HR) for the risk of death with trastuzumab compared with observation alone was 0.66. The risk for disease-free survival was assumed at 0.64 in the trastuzumab group compared to the control group. Results also showed that 1 year of treatment has a significant overall survival benefit after a median follow-up of 2 years as compared to no trastuzumab. In accordance, in our case study, patients were treated with trastuzumab during the first year modeled. Transition probabilities from PF to P used in the HERA study were directly taken into our case study [6], whereas for the transition probability from PF to death (D), data from the Dutch Central Bureau of Statistics (CBS) was used. Mortality transition probabilities were based on the assumption that the probabilities to die during PF equal general mortality rates for women in the same age classes [13].

For the late stage, data such as annual transition probabilities were based on Marty et al. (2005), where first line treatment of trastuzumab plus docetaxel was compared to docetaxel alone in patients with HER2+ metastatic breast cancer [7]. In this specific study, patients were randomly assigned to six cycles of docetaxel every 3 weeks, with or without trastuzumab until disease progression. Results showed that trastuzumab plus docetaxel was significantly superior to docetaxel alone in terms of overall response rate (61% *v* 34%), overall survival (median at 31.2 *v* 22.7 months), time to disease progression (median at 11.7 *v* 6.1 months), time to treatment failure (median at 9.8 *v* 5.3 months) and duration of response (median at 11.7 *v* 5.7 months), with marginal additional toxicity. In the late stage of our case study, patients were assumed to be treated until death [7]

In the Markov model, transition probabilities were used for early and late stage trastuzumab treatment and SOC. In both stages, trastuzumab was assigned a positive effect on health, as transition probabilities from PF to P are lower in the trastuzumab group compared to SOC. The transition probability from P to D also decreases when adding trastuzumab to the treatment regimen.

In the case study, all costs were based on the Dutch treatment guideline for trastuzumab [12]. For the adjuvant stage, patients receiving trastuzumab were assumed having a 3-weeks regimen after finishing the adjuvant chemotherapy. CVZ assumed an average Dutch treatment period of 39 weeks with 6.67mg/kg for the adjuvant stage, resulting in a cumulative dosage of trastuzumab after 13 administrations of 5522 mg [12]. With a price of €706 inclusive VAT per 150 mg [12], total costs for one year of trastuzumab treatment result at €26,000. Trastuzumab is injected intravenously in the hospital. According to the manufacturer, administration costs are €4090 for one year [11]. Trastuzumab is known for cardiac toxicity, so heart monitoring was assumed necessary [12]. The total costs in this respect are €1120, according to the manufacturer [12]. For the metastatic phase the average survival rate of 31 months amounted to drug costs of €10,400 based on a weekly dosage at 2.67mg/kg of bodyweight.

According to the CVZ report, adjuvant related health state costs were assumed at €5230 per year for the progression-free state. Metastatic health state costs were assumed at €55,800 for the first year and €12,800 for the second year, the difference being based on stopping the treatment [12]. Docetaxel costs would merely cancel each other out when comparing the groups with and without trastuzumab, therefore they are not taken into account. For simplicity, metastatic health state costs were calculated based on the survival rate and accompanying monthly costs, resulting in metastatic health state costs of (12 months x €4.650 + 19 months x €1067/31*12 =) €29,500 per year. Table 1 shows a comprehensive overview of the data used for the illustrative case study of trastuzumab in early and late stage treatment.

**Table 1:** Overview of parameters in early and late stage HER2+ breast cancer treatment with trastuzumab.

Aspect	Early stage		Late stage	
**Patient population**	HER2+ breast cancer		HER2+ metastatic breast cancer	
**Comparator**	Observation alone		Docetaxel	
**Cycles**	Administered every 3 weeks for1 or 2 years		Six cycles of trastuzumab + docetaxel or docetaxel every 3 weeks	
**Utilities**				
-**P**	0.65		0.65	
-**PF**	0.815			
**Costs (per year)**				
-**Trastuzumab treatment**	€26,000		€10,400	
-**Heart monitoring**	€1,120		€1,120	
-**Health state costs**	€5,230		€29,500	
**Transition Probability**	Early Trastuzumab	Early SOC	Late Trastuzumab	Late SOC
**PF → P**	0.0999	0.1357	-	-
**P → D**	0.716	0.727	0.2306	0.2952
**PF → D**	Age-dependent	Age-dependent	-	-
**PF**	0.8978	0.862	-	-
**P**	0.284	0.273	0.7694	0.7048

PF: Progression-free survival, P: Progression, D: Death, PF-> P: Progression-free to progression, PF-> D: Progression-free to death, P->D: Progression to death.

Effects, measured in QALYs gained, and costs used were discounted according to the Dutch guidelines: 1.5% per year for QALYs and 4% per year for costs [11]. All (incremental) costs in this study were adjusted for inflation to 2014 prices [14]. All in all, except with some slight fine tunings and updates, we basically recreated the manufacturer’s submission to the Dutch CVZ (currently ZiNL) after 4 years (t = 4) of conditional reimbursement with evidence development [12].

## Results

### Review

An overview of the drugs identified and their corresponding ICERs for early and late stages are reported in Table 2, with all ICERs being expressed in 2014 price levels. Only few consistent pairs of assessments could actually be found.

**Table 2:** Overview of ICERs for the adjuvant and metastatic phases (€ per QALY; price levels: 2014); sources: SMC and CVZ/ZiNL.

Aspect	Cetuximab	Bortezomib	Bosutinib
**Indication**			
-**Early**	Locally advanced squamous cell cancer of the head and neck	Induction treatment of adult patients with previously untreated multiple myeloma	Treatment of adult patients with chronic phase Ph+ CML
-**Late**	Advanced metastatic squamous cell cancer of the head and neck	Monotherapy for the treatment of progressive multiple myeloma in patients who have received at least one prior therapy and who have already undergone or are unsuitable for bone marrow transplantation	Treatment of adult patients with blast phase Ph+ CML
**Comparator**			
-**Early**	RT + Cetuximab vs RT	Bortezomib+TD vs TD	Bosutinib vs H/SCT
-**Late**	C/C&F+cetuximab vs C/C&F	Bortezomib vs dexamethosone	Bosutinib vs H/SCT
**Incremental costs**			
**Early**	€10,400-€12,900	€28,750	€125.000
**Late**	€21,100	€27,700	€44.800
**Incremental QALYs**			
**Early**	0.94–1.08	1.04	3.6–4.8
**Late**	0.14	0.64–0.7	0.6
**ICER (€/QALY)**			
**Early**	€11,100**-€12,000*	€27,600**	€26,200**-€34,700**
**Late**	€151,000*	€39,900*-€43,300**	€72,900**
**Potential explaining factor for difference in early vs late ICER**	Higher costs and lower QALYs in late stage	Lower QALYs in late stage	Bigger difference between costs and QALYs for late stage

* = CVZ/ZiNL;

** = SMC;

RT = RadioTherapy; TD = thalidomide and dexamethasone; H = Hydroxicarbamide; SCT = Stem cell transplantation; Ph+-CML = Philadelphia chromosome positive chronic myelogenous leukaemia; C/C&F = cisplatin or carboplatin and fluorouracil.

### Cetuximab

Cetuximab is a monoclonal antibody, which has been approved for use with concomitant radiotherapy in patients with locally advanced squamous cell carcinoma of the head and neck (SCCHN). A study for the SMC analyzed cost-utility for the adjuvant phase using a statistical “cure” model based on a phase 3, open-label randomized controlled trial [15] to estimate various survival rates and costs [16]. The comparison made involved cetuximab and radiotherapy versus radiotherapy alone. Incremental cost-effectiveness was €11,100/QALY at 0.94 QALY’s gained per patient treated with cetuximab. For the Netherlands, CVZ had assessed the dossier submitted by the manufacturer on cetuximab for that same indication [17]. Based on a very similar model being applied for Scotland, estimated cost-effectiveness for the Dutch situation was €12,000/QALY at 1.08 QALYs per patient gained treated with cetuximab; i.e., fully in line with the estimates for the SMC.

Similarly, CVZ assessed the submitted file for cetuximab in the metastatic phase for the Dutch setting [18]. Notably, the exact indication investigated referred to metastatic and/or recurrent SCCHN. The Markov model developed was based on the EXTREME clinical trial [19], inclusive mapping of EORTC scores in the trial on utilities. The comparison analyzed involved cisplatin or carboplatin and fluorouracil (C/C&F) and cetuximab versus C/C&F only. Incremental cost-effectiveness was €151,000/QALY at 0.14 QALYs gained per patient treated with cetuximab. Compared to early stage, the late stage ICER is a factor 12 higher.

### Bortezomib

Bortezomib is a proteasome inhibitor and is used to treat multiple myeloma. A cost-utility analysis was done for the SMC on data from the phase 3 PETHEMA/GEM trial, comparing bortezomib + standard treatment to standard treatment of thalidomide + dexamethason in early multiple myeloma [10]. A Markov model was developed with 3 health states, and QALYs and costs based on the literature. Notably, the incremental effects were 1.04 QALYs per patient, with corresponding incremental costs at €28,750. This resulted in an ICER of €27,600/QALY. The sensitivity analyses showed that the ICER varied between €23,000 and €38,200 when mortality probabilities were changed by +/- 20%. Other variables, such as the time-horizon chosen and an alternative post-stem cell transplantation survival resulted in ICERs of €33,500 and €24,000, respectively. Ergo, ICERs were quite robust for bortezomib in early multiple myeloma.

Bortezomib can alternatively be used as monotherapy in the treatment of progressive metastatic multiple myeloma in patients who already had previous therapy and/or bone marrow transplantation. In a study for the SMC, bortezomib monotherapy was compared to high-dose of dexamethason in patients in this late stage multiple myeloma in patients who already experienced a first relapse [11]. Data from the bortezomib registration trial was complemented with literature to design the Markov model. The incremental effects were 0.64 QALYs per patient, while incremental costs were €27,700. This resulted in an ICER of €43,100/QALY. Sensitivity analysis showed that in 32% of all cases the ICER was <€42,500/QALY. According to the underlying clinical trial, some patients needed more than the standard 24 doses of bortezomib, but that did not increase the ICER significantly. Similarly, CVZ compared bortezomib to thalidomide + dexamethason in a similar patient population [20]. The incremental effects were 0.7 QALYs per patient, while incremental costs were €27,800. This resulted in an ICER of €39,900/QALY, which is similar to results from SMC. Compared to early stage, the late stage ICER is a factor 1.5 higher.

### Bosutinib

A study for the SMC focused on the cost-utility of bosutinib for Philadelphia-chromosome positive chronic myeloid leukemia (Ph+ CML), in chronic acceleration and the blast phases of the disease [21]. Notably, the amount of affected red blood cells increases with every phase, with the chronic phases considered here as adjuvant or early stage and blast as metastatic or late phase. In the study, bosutinib is compared to hydroxycarbamine and stem cell transplantation in adult patients who were previously treated with one or more tyrosin kinase inhibitors and who were not eligible for treatment with imatinib, nilotinib and dasatinib. Markov modeling was used, utilities were derived from the National Institute of health and Clinical Excellence (NICE) study on imatinib and costs were also based on NICE-analyses.

In the early stage, incremental costs varied from savings (stem cell) to approximately €125,000 per patient and QALYs from 3.6 to 4.8. In the late stage, these numbers were up to approximately €44,800 and to 0.6, respectively. In the end, cost-effectiveness ratios were far higher in the late than in the early stage; i.e. up to over €72,900/QALY versus cost saving potentials. This resulted in the late stage ICER being at least a factor 3 higher than early stage.

### Case study trastuzumab

As mentioned, we basically recreated the manufacturer’s submission for trastuzumab at t = 4 for the Netherlands [12]. Trastuzumab lowers all transition probabilities in both early and late stage, making it an effective treatment for HER2-positive breast cancer. Compared to late stage, the early stage treatment lowers the probability to transition to next state more, making it relatively more effective. The highest costs and lowest health gains were both found after treating patients with trastuzumab in late stage.

After discounting, early stage incremental costs were €25,000 with 2.7 QALYs gained per patient, resulting in an ICER of €9,250/QALY. Late stage incremental costs were €123,000 with 1.3 QALYs gained per patient, resulting in an ICER of €94,600/QALY. For example, using a potential threshold of €80,000/QALY [4], the early stage would be deemed cost-effective, while the late stage is a factor 10 more expensive, lifting it over the assumed threshold and potentially considered not cost-effective.

## Discussion

We searched for analyses performed by one and the same authority for oncological drugs in the early and late stages of disease, but these are still rare. Exactly these studies could produce reliable connections between both stages. Still, scarce studies that were found, often slightly differed in patient population, clinical data, perspective, costs, discounting utilities, frequencies of treatment and parameters of survival. Sometimes CVZ or SMC offered only early or late stage cost-utility analyses. In that case, in addition to using SMC and CVZ as sources, additional literature was identified in PUBMED and subsequently [22], ideally for the same or comparable countries.

Treatments in the late stage were found to be more expensive by a factor ranging from 1.5 to 12. Notably, at a potential threshold of €80,000/QALY [4], cetuximab and bosutinib can be considered cost-effective in the early stage, while late-stage ICERs may be considered not cost-effective. Relatively low ICERs in the early stage are caused by a major potential still to gain QALYs and relatively low medical costs, as patients are still relatively healthy and a likelihood for cure still exists. Subsequently, a high ICER in the late phase may be related to a lower potential to win QALYs, as overall survival is relatively short in the late stage. Furthermore, costs in the metastatic phase are often higher, as more frequent use of health care is usually needed to treat metastatic patients. Our results illustrate that in early stage, QALYs gained range from 0.94 to 4.8 QALYs, while in late stage QALYs gained ranged from 0.14 to 0.7. Also, in early stage, incremental costs ranged from €11,100 to €125,000, while in late stage incremental costs ranged from €44,800 to €1,079,000. It should be noted that all adjuvant trials used for the cost-effectiveness analyses are based on a relatively short follow-up time of 1–3 years. Therefore it seems possible that hazard ratios could be lower after a longer period of time, which could impact the ICER negatively.

Bortezomib could be considered cost-effective in both early and late stage. In most cases, costs for metastatic patients are much higher compared to adjuvant patients, as more intensive (and expensive) therapy is needed. Bortezomib is the exception, as previously untreated adjuvant patients get 4 cycles of therapy, while the metastatic patients, who presumably already had their progressive myeloma treated, get “only” 2 cycles of therapy [10, 11]. The late phase ICER (€43,100) is still higher than in the early phase (€27,770), because there are less QALYs to be gained in the late phase (0.64 vs 1.04). Also, additional costs like GP visits and extra controls drove the metastatic costs further up, resulting in a higher ICER. In addition, a study for NICE for metastatic patients receiving 3 cycles of therapy resulted in €53,600/QALY [23], very similar to the SMC study.

In addition, for cetuximab a published study was identified that specifies cost-utility for Belgium and the UK in early; i.e., the adjuvant phase [24]. Notably, the comparison made involved cetuximab and radiotherapy versus radiotherapy alone. Local costs were used in both countries, whereas a uniform discount rate at 3.5% was applied. The incremental effects were 1.08 QALYs for Belgium and the UK, while incremental costs were €11,400 and €12,900, respectively. Treatment with cetuximab plus radiotherapy won 3.96 QALYs on average, compared to 2.88 QALYs when treating with radiotherapy only. Treatment with cetuximab was grossly €10,000 more expensive than radiotherapy alone, with similar patterns for both countries. Similarly, NICE did the same study on cetuximab in the early stage, resulting in an ICER of €10,200/QALY [25], which is similar to the previously found ICER of €11,100/QALY [16].

Furthermore, a Canadian study looked at the cost-effectiveness of cetuximab + platinum-based chemotherapy of patients with recurring or metastasized SCCHN in the primary care [26]. Data was taken from a phase 3 trial on patients with SCCHN. Using a Markov model, variables as costs, side effects, QALY’s etc were taken in to account when comparing the effects of cetuximab + standard with only the standard treatment. Gaps in the phase 3 trial were filled with information from the manufacturer or literature. The discount rate was 5% for effects and costs. The assumption was made that cetuximab did not influence the quality of life in any way. The incremental effects were 0.093 QALY’s, while incremental costs were €28,300. This resulted in an ICER of €303,000/QALY. A sensitivity analysis showed that the ICER is most sensitive to the cetuximab price and the risk of progression. It also showed that the model is not sensitive for a change in the severity of side effects, its frequencies and their subsequent costs. The ICER remained above €215,000. Similarly, NICE did a study on cetuximab in the late stage, resulting in an ICER of €155,000/QALY [27].

CVZ analyzed trastuzumab according to the HERA trial in early stage breast cancer for the Dutch situation, resulting in an ICER of €16,700/QALY, very similar to our case study’s early stage estimate [12]. For late stage trastuzumab treatment, a Norwegian cost-effectiveness analysis by Marty et al. (2005) resulted in incremental costs of €54,952 and ICERs ranging from €78,500 to €183,200 per life-year gained, adding between 0.3 and 0.7 years [28]. Only effects were discounted with 5%, as all costs occurred in the first year. This is on par with the late stage ICER from our case study of €94,600/QALY, at 1.3 QALYs gained per patient.

Notably, more favorable transition probabilities, lower treatment costs and more QALYs saved are factors that contribute to a more favorable ICER in use of drugs in early stage oncology. For reimbursement agencies, this potential trend in early vs late stage cost-effectiveness could mean that ICERs in the late stage should be considered taking into account the anticipation that ICERs tend to be lower after the same oncological drug is submitted later on for early stage treatment. Pharmaceutical companies could anticipate accordingly by looking at different price/volume situations and patient access schemes.

From our case study, trastuzumab treatment would be cost-effective only in the early stage using a threshold of €80,000/QALY. Late stage cost-effectiveness is a factor 10 more unfavourable than early stage. By definition, this is related to differences in incremental costs and QALYs gained between early and late stage. The case study resulted in lower incremental costs for early stage (€25,000 vs €123,000 in late stage), with lower progression-free health state costs being the most influential factor. Also, more QALYs gained (2.7 vs 1.3 in late phase) contributed to the early-stage treatment being considered cost-effective, while late-stage treatment is not.

Another potential factor for differences in ICERs relates to transition probabilities. Early stage treatment lowers the transition probability to progress by 26%. The comparable late stage transition probability to death decreases by 22%, rendering trastuzumab treatment more effective in the early stage compared to late stage. This further explains why early treatment is more cost-effective than late stage treatment. We note that one limitation in our case study is that in the early stage trastuzumab is considered alone, while in the late stage, trastuzumab+docetaxel is considered.

In general, using for example a threshold of €80,000/QALY [4], early stage treatment may often be considered cost-effective while late stage is often not. Authorities should be aware of this when assessing oncological drugs and interpreting the corresponding cost-effectiveness ratios. Even though a pattern is identified in our study, further research is needed to keep confirming our findings.

## Conclusions

Through review and case study, we found that the ICERs of drugs used in different stages of cancer, are higher (i.e. more unfavorable) in the metastatic phase than in the adjuvant phase or early vs. late stages. Reasons identified were higher costs because of a more intensive treatment, more drug use and subsequently more intensive monitoring in the late stages of cancer. Moreover, in the early stages of cancer, a higher potential for QALY gains exist, compared to the metastatic phase. Notably, compared to early-stage ICERs, late-stage ICERs were found to be 1.5 to 12 times higher. Our case study on trastuzumab further confirmed our findings.

Ergo, oncological drugs in the respective stages of early and late cancer may hugely differentiate in the respective ICERs. Authorities often first-time encounter with the late-stage indication of these drugs, with relatively unfavourable ICERs. Not always may they sufficiently differentiate between early and late stages in their interpretation of results and not envision the future more cost-effective application of the same drug in the early stage. Notably, an approval in the early setting frequently leads to a shift in use of the drug from the late to the early setting rather than that the drug is being used in both settings. Such future outlooks illustrate that exact differentiation between both stages is crucial and may help authorities to better allocate scarce resources in oncology and enhance access to patients to these potentially life-savings drugs.

## Supporting Information

S1 Fig Early vs Late model trastuzumab. (XLSX)
